# Hollow Carbon and MXene Dual‐Reinforced MoS_2_ with Enlarged Interlayers for High‐Rate and High‐Capacity Sodium Storage Systems

**DOI:** 10.1002/advs.202400364

**Published:** 2024-01-22

**Authors:** Hanqing Pan, Yan Huang, Xinnuo Cen, Ming Zhang, Jianhua Hou, Chao Wu, Yuhai Dou, Bing Sun, Ying Wang, Binwei Zhang, Lei Zhang

**Affiliations:** ^1^ Jiangsu Key Laboratory of Green Synthetic Chemistry for Functional Materials School of Chemistry & Materials Science Jiangsu Normal University Xuzhou Jiangsu 221116 P. R. China; ^2^ College of Environmental Science and Engineering Yangzhou University Yangzhou Jiangsu 225009 P. R. China; ^3^ Institute of Energy Materials Science University of Shanghai for Science and Technology Shanghai 200093 P. R. China; ^4^ Centre for Clean Energy Technology School of Mathematical and Physical Sciences Faculty of Science University of Technology Sydney Ultimo NSW 2007 Australia; ^5^ School of Chemistry and Chemical Engineering Chongqing University Chongqing 401331 P. R. China; ^6^ Center of Advanced Electrochemical Energy Institute of Advanced Interdisciplinary Studies Chongqing University Chongqing 401331 P. R. China; ^7^ Centre for Catalysis and Clean Energy Gold Coast Campus Griffith University Gold Coast QLD 4222 Australia

**Keywords:** electrodes, hollow carbon, MoS_2_, MXene, sodium storage systems

## Abstract

Sodium‐ion batteries (SIBs) and sodium‐ion capacitors (SICs) are promising candidates for cost‐effective and large‐scale energy storage devices. However, sluggish kinetics and low capacity of traditional anode materials inhibit their practical applications. Herein, a novel design featuring a layer‐expanded MoS_2_ is presented that dual‐reinforced by hollow N, P‐codoped carbon as the inner supporter and surface groups abundant MXene as the outer supporter, resulting in a cross‐linked robust composite (NPC@MoS_2_/MXene). The hollow N, P‐codoped carbon effectively prevents agglomeration of MoS_2_ layers and facilitates shorter distances between the electrolyte and electrode. The conductive MXene outer surface envelops the NPC@MoS_2_ units inside, creating interconnected channels that enable efficient charge transfer and diffusion, ensuring rapid kinetics and enhanced electrode utilization. It exhibits a high reversible capacity of 453 mAh g^−1^, remarkable cycling stability, and exceptional rate capability with 54% capacity retention when the current density increases from 100 to 5000 mA g^−1^ toward SIBs. The kinetic mechanism studies reveal that the NPC@MoS_2_/MXene demonstrates a pseudocapacitance dominated hybrid sodiation/desodiation process. Coupled with active carbon (AC), the NPC@MoS_2_/MXene//AC SICs achieve both high energy density of 136 Wh kg^−1^ at 254 W kg^−1^ and high‐power density of 5940 W kg^−1^ at 27 Wh g^−1^, maintaining excellent stability.

## Graphical abstract

A cross‐linked NPC@MoS_2_/MXene of layer‐expanded MoS_2_ that dual‐reinforced by hollow N, P‐codoped carbon (NPC) as the inner supporter and surface groups abundant MXene as the outer supporter is developed for sodium storage systems, which achieves high energy density of 136 Wh kg^−1^ at 254 W kg^−1^ and high‐power density of 5940 W kg^−1^ at 27 Wh g^−1^.

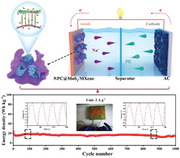

## 1 Introduction

In the current era, the demand for sustainable and clean energy sources is increasing rapidly, necessitating the development of high‐performance energy storage devices such as batteries and hybrid supercapacitors. Among the various types of energy storage systems, sodium‐ion batteries (SIBs) and sodium‐ion capacitors (SICs) have emerged as promising alternatives to lithium‐ion batteries due to the abundance and low cost of sodium resources. However, the large Na^+^ radius and sluggish Na^+^ diffusion kinetics have limited the cycling stability and energy density of SIBs, especially at high current densities. This challenge became more urgent in SICs, which are composed of a battery‐type anode and a capacitor‐type cathode. The mismatch of reaction kinetics between an intercalation‐type anode and an electric double‐layer capacitor‐type (EDLC) cathode has become the primary obstacle for SICs.^[^ ^1^ ^]^ Thus, anode materials with fast sodium storage kinetics and robust structure to tolerate the repeated sodiation/desodiation process have become a key limitation in developing high‐performance SIBs and SICs.

Generally, layered materials, such as manganese oxides^[^ ^2^ ^]^ and layered Ti‐based materials,^[^ ^3^ ^]^ have been regarded as promising anodes for both SIBs and SICs due to the facile Na^+^ transportation through 2D diffusion channels. Among them, molybdenum disulfide (MoS_2_), with an insertion and conversion combined Na^+^ storage mechanism, stands out owing to its large interlayer spacing, weak van der Waals forces, and high theoretical capacity (675 mAh g^−1^).^[^ ^4^ ^]^ However, the sodiation/desodiation process of MoS_2_ is accompanied by severe volume change, leading to a degradation of its layered structure further resulting in a rapid capacity decay. Besides, the poor electrical conductivity of MoS_2_ has limited its electrochemical reaction kinetics. Expanding the interlayer distance of MoS_2_ layers and combining MoS_2_ with conductive materials are the most useful approaches to improve the sodium storage performances of MoS_2_.^[^ ^5^ ^]^ To date, plenty of works have focused on designing hybrid MoS_2_/C composites with enlarged MoS_2_ interlayers. For example, Han et al. reported a charge‐driven interlayer expansion strategy to synthesize MoS_2_/C composites which showed excellent electrochemical performance for SIBs.^[^ ^6^ ^]^ However, a nonnegligible issue of these layer expanded MoS_2_/C composites is the unsatisfied electrochemical kinetics due to extended charge transfer distance.^[^ ^7^ ^]^ Therefore, introducing another high conductive skeleton material to MoS_2_/C hybrids is a promising strategy to further improve the sodium storage performances.

MXene, a graphene‐like 2D transition metal carbide with metal conductivity and remarkable mechanical flexibility, has attracted significant attention in high‐efficiency energy storage and conversion systems.^[^ ^8^ ^]^ Researchers have investigated the potential of combining MoS_2_ with MXene to create hybrid electrodes. For instance, Wang et al. reported a MoS_2_/MXene heterostructure that confirmed the positive effects of MXene on improving the electrochemical performance of MoS_2_.^[^ ^9^ ^]^ It should be noted that research about the MoS_2_, carbon, and MXene hybrid electrode is just at the beginning and most of the reported samples enveloped the MXene inside.^[^ ^10^ ^]^ However, the rich functional groups in MXene make it perfect to be exposed on the surface, which can not only absorb extra Na^+^ but also boost high pseudocapacitive response.^[^ ^8^, ^11^ ^]^ Generally, pseudocapaictive electrodes can store energy through battery‐like faradaic reactions, which undergoes rapid charge/discharge processes comparable to those of EDLCs cathodes. Therefore, endowing the battery‐type anodes with high pseudocapacitive response is promising for high‐performance Na^+^ storage, especially for the SICs.

In this study, we developed a interesting structure of layer‐expanded MoS_2_ that dual‐reinforced by hollow N, P‐codoped carbon (NPC) as the inner supporter and surface groups abundant MXene as the outer supporter, forming a cross‐linked electrode of NPC@MoS_2_/MXene. The interlayer distance of MoS_2_ (≈0.98 nm) is enlarged by in situ intercalation of carbon into the MoS_2_ gallery, which provides adequate channels for Na^+^ diffusion. The hollow NPC functions as an internal matrix to guide the growth of MoS_2_, simultaneously endows stronger interconnection between MoS_2_ and the carbon matrix to protect the structure from collapse. To further strengthen the structure stability and facilitate the charge transfer/ion diffusion kinetics, MXene layers were used as outer‐supporter to wrap the NPC@MoS_2_ units inside. Boosted by the synergistic effects among hollow NPC, MoS_2_, and MXene the prepared NPC@MoS_2_/MXene exhibited high reversible capacity (453 mAh g^−1^) and unparalleled rate capability (with 54% capacity retention as the current density increases from 100 to 5000 mA g^−1^) than that of NPC@MoS_2_, MoS_2_/MXene and other reported MoS_2_‐based electrodes for SIBs. Mechanism investigations reveal that the exposure of the MXene surface is crucial for achieving improved Na^+^ storage performance. This exposure not only enhances electrochemical kinetics but also facilitates a high pseudocapacitance behavior. When further applied the NPC@MoS_2_/MXene for SICs, the assembled NPC@MoS_2_/MXene//AC full cell capacitors showed both high‐energy and high‐power densities with good cycling stability.

## 2 Results and Discussion

**Scheme 1:**
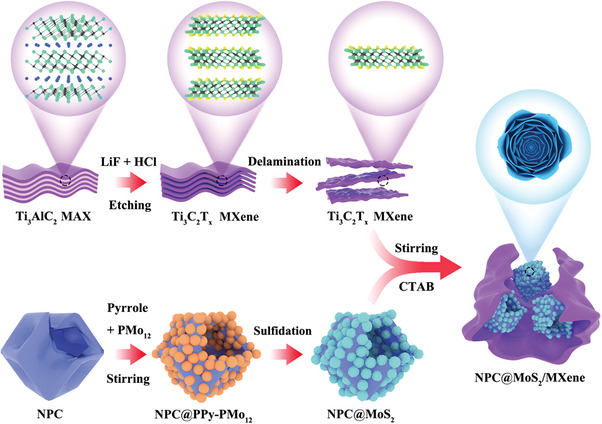
Illustration for the synthesis process of the NPC@MoS_2_/MXene.

The synthesis process of the hollow NPC@MoS_2_/MXene composite is illustrated in **Scheme** **1**. Initially, hollow NPC (derived form ZIF‐8, structural information in Figures S1 and S2, Supporting Information) was modified by Polyvinyl Pyrrolidone (PVP) and employed as the “inner‐supporter” to grow MoS_2_. Subsequently, a mixture of pyrrole monomer and phosphomolybdic acid (PMo_12_) that served as both a Mo source and an oxidant to trigger the polymerization of pyrrole was added to the PVP‐modified NPC suspension. The in situ formation of polypyrrole (PPy) encapsulated the PMo_12_ inside and precipitated on the NPC surface, leading to the formation of NPC@PPy‐PMo_12_ (confirmed by the FTIR spectra in Figure S3, Supporting Information). The subsequent annealing process involved the carbonization of PPy and the phase transition of PMo_12_ into MoS_2_, thereby forming hollow NPC@MoS_2_ units. Finally, the exfoliated Ti_3_C_2_T_x_ MXene (structural information in Figure S4, Supporting Information) was adsorbed onto the surfaces of hollow NPC@MoS_2_ through electrostatic attraction (evidenced by the Zeta potential test in Figure S5, Supporting Information) and acted as the “out‐supporter”. For comparison, single NPC or MXene reinforced NPC@MoS_2_ and MoS_2_/MXene composites were also synthesized using a similar method, without the addition of MXene or NPC, respectively.

**Figure 1:**
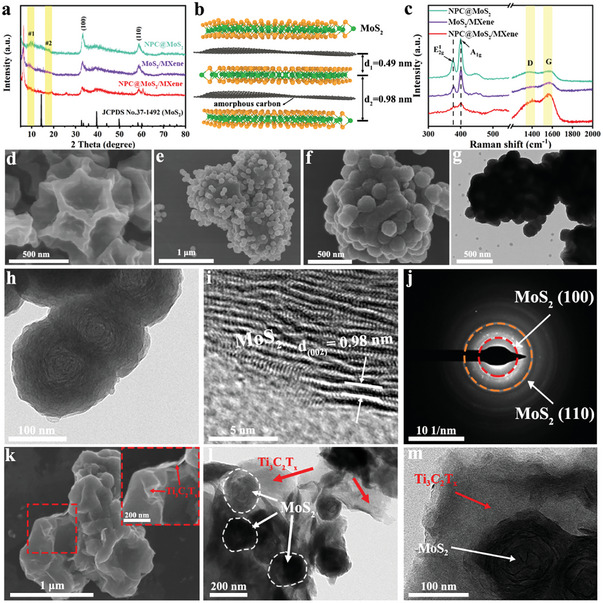
a) XRD patterns of the NPC@MoS_2_, MoS_2_/MXene and NPC@MoS_2_/MXene, b) crystal structure illustration of the NPC@MoS_2_/MXene, c) Raman curves of the NPC@MoS_2_, MoS_2_/MXene and NPC@MoS_2_/MXene. SEM images of the d) hollow NPC, e) NPC@PPy‐PMo_12_, and f) NPC@MoS_2_. g,h) TEM, i) HR‐TEM, and j) SAED images of the NPC@MoS_2_, k) SEM image of NPC@MoS_2_/MXene, l,m) TEM images of the NPC@MoS_2_/MXene in different magnifications.

The X‐ray diffraction (XRD) patterns of the synthesized NPC@MoS_2_, MoS_2_/MXene, and NPC@MoS_2_/MXene samples were presented in **Figure** **1**a. Diffraction peaks corresponding to the (100) and (110) planes of MoS_2_ (JCPDS 37–1492) were clearly observed in all synthesized samples, indicating the successful sulfidation of the PPy‐PMo_12_. Notably, the sharp diffraction peak of the (002) plane that was assigned to the restacking of MoS_2_ layers was absent, suggesting a single/fewer layered structure of the synthesized MoS_2_.^[^ ^12^ ^]^ This implied that the presence of PPy‐derived carbon likely plays a crucial role in preventing the restacking of MoS_2_ layers. A closer examination of the XRD patterns revealed two new peaks at 2*θ* = 17.8°(labeled as #1) and 9.0° (labeled as #2), indicating the expanded interlayer spacing of MoS_2_. According to the Bragg's law, the *d*‐spacing of peak #1 and peak #2 is 0.49 and 0.98 nm, respectively. Considering that the *d*‐spacing of MoS_2_ and carbon layers is 0.62 and 0.34 nm, respectively, it is highly likely that the PPy‐derived amorphous carbon inserts into the MoS_2_ gallery (as shown in Figure 1b). Peak #1 corresponded to the distance from carbon to the MoS_2_ layer, while peak #2 corresponded to the distance between adjacent MoS_2_ layers with carbon.^[^ ^12a^ ^]^ To confirm the positive impact of the PPy‐derived amorphous carbon in expanding the interlayer distance of MoS_2_ sheets, we examined X‐ray diffraction (XRD) and high‐resolution transmission electron microscopy (HR‐TEM) images of the MoS_2_ derived from pure PPy‐PMo_12_. The XRD pattern in Figure S6 (Supporting Information) exhibited a similar property of layer expansion as observed in these hybrid materials, and the HR‐TEM images in Figure S7 (Supporting Information) confirmed a substantial interlayer distance of 0.98 nm between MoS_2_ layers. This expanded interlayer distance of MoS_2_ provides favorable diffusion channels for the rapid insertion and extraction of Na^+^ ions.^[^ ^6^ ^]^ In comparison to the NPC@MoS_2_, the intensity of the MoS_2_ peaks in MoS_2_/MXene and NPC@MoS_2_/MXene was relatively low, while a typical peak for Ti_3_C_2_T_x_ MXene (002) at 6.2° was observed, indicating the successful encapsulation of MXene on their outer surface. Additional structural information about the synthesized samples was obtained through Raman spectra. As shown in Figure 1c, all three samples exhibited the E1_2g_ and A_1g_ vibration modes of MoS_2_.^[^ ^13^ ^]^ The most intense peaks for MoS_2_ were observed in the NPC@MoS_2_ sample, agreed with its exposed MoS_2_ surface. Furthermore, two additional peaks at 1385 cm^−1^ and 1562 cm^−1^ were observed, corresponding to the disorder‐induced D‐band and the *sp^2^* hybridized graphitic G‐band of carbon, respectively.^[^ ^12b^ ^]^ Compared with pure MXene (a negligible peak for the D band was observed, as shown in Figure S8, Supporting Information), the increased D bond of MoS_2_/MXene confirmed the successful insertion of PPy‐derived disordered carbon into the MoS_2_ gallery. The highest I_D_/I_G_ ratio was exhibited in the NPC@MoS_2_ sample (0.95), indicating the defect‐rich structure of the hollow NPC. After further being wrapped by MXene, the NPC@MoS_2_/MXene sample showed a moderate I_D_/I_G_ ratio of 0.89.

Scanning electron microscopy (SEM) and TEM images were utilized to observe the morphological and structural characteristics of the samples. The “inner‐supporter” of hollow NPC exhibited a polyhedral structure with a uniform size distribution of ≈500 nm (Figure S9, Supporting Information; Figure 1d). After the PVP modification, the PPy‐PMo_12_ spheres with a diameter of ≈47 nm were observed to anchor on the surface of the hollow NPC, forming the NPC@PPy‐PMo_12_ intermediate (Figure S10, Supporting Information; Figure 1e). Following the annealing process, the resulting hollow NPC@MoS_2_ maintained a similar morphology, with numerous spheres loaded onto the polyhedral structure (Figure 1f). The highly wrinkled surface of the MoS_2_ spheres indicated a possible layered structure. The TEM image in Figure 1g corroborated the findings from the SEM analysis. A closer examination in Figure 1h revealed that the spheres were composed of ultrathin nanosheets, forming an onion‐like structure. The HR‐TEM image in Figure 1i revealed a significantly reduced stacking of MoS_2_ layers, indicating the presence of only a few layers. Moreover, the measured *d*‐spacing of the MoS_2_ layers (0.98 nm) aligned with the XRD results and confirmed the insertion of the carbon layer into the MoS_2_ gallery. The selected area electron diffraction (SAED) pattern in Figure 1j confirmed the polycrystalline nature of the MoS_2_ onion‐like sphere. Elemental mapping images in Figure S11 (Supporting Information) demonstrated the uniform distribution of Mo, S, C, N, and P in the NPC@MoS_2_, with elemental contents of 48%, 36%, 11%, 1%, and 4% for Mo, S, C, N, and P, respectively. To further analyze the composition of the NPC@MoS_2_, thermogravimetric analysis (TGA) was conducted. The first weight loss in Figure S12 (Supporting Information) was attributed to the evaporation of adsorbed water, and the second weight loss from 300 to 700 °C was ascribed to the oxidation of MoS_2_ to MoO_3_ and the combustion of carbon. Consequently, the mass content of NPC and MoS_2_ in the NPC@MoS_2_ was calculated to be 13.5% and 86.5%, respectively, which aligns with the SEM mapping results. Following the addition of MXene, which served as an “outer supporter” to enhance structural integrity and electronic conductivity, the final NPC@MoS_2_/MXene composite was obtained. The SEM image in Figure 1k revealed the successful wrapping of MXene layers around the NPC@MoS_2_ units. TEM images at the edge further confirmed the strong interconnection between the MXene layer and the NPC@MoS_2_, as evident in Figure 1m.

**Figure 2:**
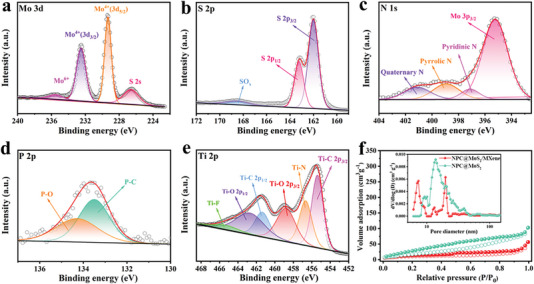
High‐resolution XPS spectrum of a) Mo 3d, b) S 2p, c) N 1s, d) P 2p, and e) Ti 2p of the NPC@MoS_2_/MXene. f) Nitrogen‐isothermals of the NPC@MoS_2_ and NPC@MoS_2_/MXene. Inset (f) is the pore size distribution plots of the NPC@MoS_2_ and the NPC@MoS_2_/MXene.

The interaction between the hollow NPC, MoS_2_ layers, and MXene was investigated using X‐ray photoelectron spectroscopy (XPS). The XPS survey in Figure S13 (Supporting Information) confirmed the presence of Mo, S, C, N, P, and Ti elements in the NPC@MoS_2_/MXene composite. High‐resolution spectra from the XPS survey were presented in **Figure** **2**. The Mo 3d XPS plot in Figure 2a exhibited two dominant peaks at 232.5 and 229.4 eV, corresponding to the Mo 3d_3/2_ and Mo 3d_5/2_ states of Mo^4+^.^[^ ^14^ ^]^ The presence of a S 2s peak at 226.6 eV confirmed the formation of the MoS_2_ state.^[^ ^15^ ^]^ Additionally, a small peak at 235.6 eV indicated surface oxidation of the MoS_2_. The successful preparation of MoS_2_ was further supported by the S 2p XPS plot in Figure 2b, which displayed two peaks at 162.2 and 163.4 eV corresponding to the Mo─S bonds in MoS_2_.^[^ ^14^, ^15^ ^]^ The broad peak at 168.4 eV was attributed to a C‐S‐O vibration, indicating the coupling interaction among carbon, MoS_2_, and the hydroxide group in MXene.^[^ ^16^ ^]^ The nitrogen doping of the hollow NPC was confirmed through the analysis of the N 1s spectra in Figure 2c, revealing characteristic peaks associated with pyridinic N, pyrrolic N, and graphitic N.^[^ ^17^ ^]^ Surprisingly, the hollow NPC was found to be doped with phosphorus (P), as evidenced by the presence of characteristic peaks corresponding to P─C and P─O bonds^[^ ^18^ ^]^ at 133.5 and 134.4 eV, as observed in Figure 2d. Previous reports have demonstrated that such N, P‐codoped carbon can introduce additional active sites for Na^+^ storage, enhance the electrode's conductivity, and facilitate the transfer of Na^+^ ions.^[^ ^18^, ^19^ ^]^ The Ti 2p XPS plot in Figure 2e displayed signals at 455.4 and 461.4 eV for the Ti‐C 2p_3/2_ and Ti‐C 2p_1/2_ bonds, 458.8 and 462.9 eV for the Ti‐O 2p_3/2_ and Ti‐O 2p_1/2_ bonds, and a singlet for the Ti‐F bond, indicating the presence of abundant terminal groups on the surface of MXene.^[^ ^9^, ^16^, ^20^ ^]^ The N_2_ absorption/desorption isotherms in Figure 2f exhibited a type‐IV pattern, suggesting that both NPC@MoS_2_ and NPC@MoS_2_/MXene possessed mesoporous structures. After the wrapping of MXene, the specific surface area of NPC@MoS_2_/MXene decreased from 74.8 to 39.6 m^2^ g^−1^ due to the relatively low surface area of MXene.^[^ ^11^ ^]^ The pore size distribution plots inset in Figure 2f confirmed the effective wrapping of MXene on the surface of NPC@MoS_2_ units. The calculated pore volume for NPC@MoS_2_/MXene was 0.159 cm^3^ g^−1^. The high pore volume and mesoporous structure of NPC@MoS_2_/MXene not only alleviate the volume change of the electrode during cycling but also facilitate better electrolyte‐electrode contact from both inside and outside, providing shorter diffusion paths for ionic and electronic transport.

**Figure 3:**
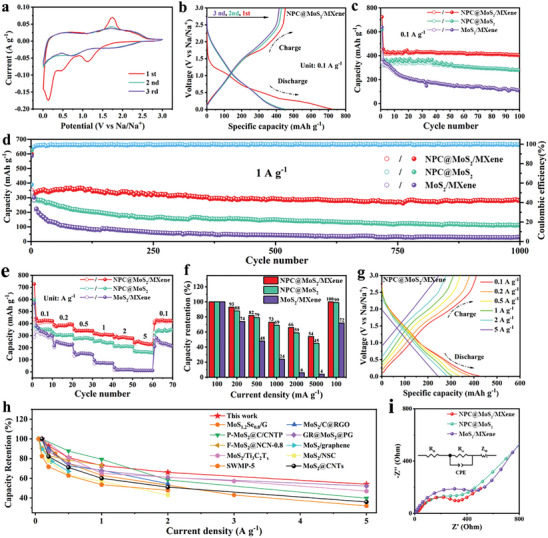
Electrochemical performances in SIBs: a) CV plots at a scan rate of 0.1 mV s^−1^ and b) charge/discharge profiles at a specific capacity of 100 mA g^−1^ of the NPC@MoS_2_/MXene; Cycling performances at c) 100 mA g^−1^, d) 1000 mA g^−1^, and e) the rate capability and f) capacity retention ratio at various current densities various from 100 to 5000 mA g^−1^ of the NPC@MoS_2_, MoS_2_/MXene and NPC@MoS_2_/MXene, respectively; g) discharge/charge profiles of the NPC@MoS_2_/MXene at various current densities; h) electrochemical performances comparison of the as‐prepared NPC@MoS_2_/MXene with other related electrodes; i) Nyquist plots of the NPC@MoS_2_, MoS_2_/MXene and NPC@MoS_2_/MXene. Inset (i) the equivalent circuit model of electrodes.

Sodium storage performance evaluations were conducted on the hollow NPC@MoS_2_/MXene composite as an anode material for SIBs, and comparisons were made with NPC@MoS_2_ and MoS_2_/MXene (SEM image in Figure S14, Supporting Information). The initial three cyclic voltammetry (CV) curves of the NPC@MoS_2_/MXene were depicted in **Figure** **3**a. During the first cathodic scan, two distinct peaks at 1.10 and 0.46 V were observed, corresponding to the insertion of Na^+^ into the MoS_2_ layers and the formation of a solid electrolyte interface (SEI) film, respectively.^[^ ^9^, ^10^ ^]^ The broad peak near 0.1 V may be attributed to the intercalation of Na^+^ into MXene and/or carbon, as well as the dispersion of Mo into Na_2_S.^[^ ^21^ ^]^ Subsequent cycles exhibited two broad peaks around 0.7 and 1.7 V, resulting from the intercalation of Na^+^ into MoS_2_, forming Na_x_MoS_2_, and subsequent conversion reactions. The anodic scans showed peaks at around 0.6 and 1.6 V, corresponding to the sequential phase transitions during the desodiation process.^[^ ^9^, ^10^ ^]^ After the first cycle, the CV curves showed good overlap, indicating the high reversibility of the NPC@MoS_2_/MXene electrode. Similar peak positions and shapes were observed in the CV curves of NPC@MoS_2_ (Figure S15a, Supporting Information) and MoS_2_/MXene (Figure S16a, Supporting Information) due to their similar chemical compositions. The CV curves of pure MXene and NPC (Figure S17, Supporting Information) exhibited capacitive‐like behavior resulting from the insertion of Na^+^ into the MXene and NPC layers. Galvanostatic discharge/charge plots of the NPC@MoS_2_/MXene in Figure 3b exhibited excellent agreement with the CV curves. The initial specific discharge/charge capacities were 723/453 mAh g^−1^, corresponding to an initial Coulombic efficiency (ICE) of 63%. The irreversible capacity loss observed during the first cycle may stem from several factors, including electrolyte decomposition, irreversible trapping of Na^+^ ions within the electrode, sluggish electrochemical kinetics of certain unprotected MoS_2_ sheets, and other irreversible side reactions occurring at the electrode interface.^[^ ^22^ ^]^ It is worth noting that the initial Coulombic Efficiency (ICE) of the NPC@MoS_2_/MXene electrode can potentially be enhanced through presodiation, optimization of battery configurations, and electrolyte improvements. At the end of the third cycle, the NPC@MoS_2_/MXene maintained a discharge/charge capacity of 432/422 mAh g^−1^, which was higher than that of NPC@MoS_2_ (357/332 mAh g^−1^, shown in Figure S15b, Supporting Information) and MoS_2_/MXene (346/321 mAh g^−1^, shown in Figure S16b, Supporting Information). It should be noted that the specific capacity was calculated based on the entire active materials. To better understanding the electrochemical performances of NPC@MoS_2_, MoS_2_/MXene and NPC@MoS_2_/MXene, the chemical composition of each sample was listed in Table S1 (Supporting Information).

The cycling stability of the electrodes was tested at a specific current density of 100 mA g^−1^. As shown in Figure 3c, the NPC@MoS_2_/MXene exhibited the highest reversible capacity during 100 cycles. It maintained a high capacity of 411 mAh g^−1^ at the end of 100 cycles, corresponding to a capacity retention of 91%. The NPC@MoS_2_ sample maintained a capacity of 281 mAh g^−1^ with a retention of 79% at the end of 100 cycles. The MoS_2_/MXene sample exhibited the lowest capacity of only 117 mAh g^−1^, with a retention as low as 32%. Typically, the specific capacity of these MoS_2_‐based electrodes primarily originates from MoS_2_, which experiences significant volume change and phase transitions during cycling, leading to structural degradation and capacity loss. Therefore, it is reasonable to deduce that the NPC plays an important role in maintaining the structural stability of MoS_2_, as evident from the significantly higher capacity retention of NPC@MoS_2_ and NPC@MoS_2_/MXene compared to the MoS_2_/MXene sample. The enhanced structural stability of these NPC‐containing samples was further confirmed by their nearly overlapping discharge/charge profiles in Figure S18 (Supporting Information). The long‐term cycling stability of these electrodes was further investigated at a higher specific current density of 1000 mA g^−1^, as shown in Figure 3d. Even after 1000 cycles at 1000 mA g^−1^, the NPC@MoS_2_/MXene still exhibited the highest reversible capacity of 279 mAh g^−1^ with a low capacity decay of only 0.021% per cycle (compared with the second cycle), outperforming the NPC@MoS_2_ and MoS_2_/MXene samples. The high CE of NPC@MoS_2_/MXene, approaching 100% after activation in the initial cycles, also demonstrated the high stability of this MXene and NPC dual‐reinforced structure of NPC@MoS_2_/MXene.

Rate capability tests were conducted on NPC@MoS_2_, MoS_2_/MXene, and NPC@MoS_2_/MXene at various current densities ranging from 100 to 5000 mA g^−1^. As shown in Figure 3e, the discharge/charge capacities of MoS_2_/MXene were comparable to those of NPC@MoS_2_ in the initial cycles. Considering the relatively low theoretical capacity of MoS_2_/MXene, due to the low capacity of MXene shown in Figure S17d (Supporting Information), it can be inferred that MXene enables higher utilization of the MoS_2_. Therefore, the best rate capability was observed in the NPC@MoS_2_/MXene sample, which combines the cycling stability provided by NPC and the enhanced utilization facilitated by MXene. High reversible capacities of 428, 393, 346, 316, and 289 mAh g^−1^ were achieved at current densities of 100, 200, 500, 1000, and 2000 mA g^−1^, respectively. Even at a higher current density of 5000 mA g^−1^, NPC@MoS_2_/MXene still retained a higher capacity of 239 mAh g^−1^. When the current density was dropped back to 100 mA g^−1^, the capacity immediately recovered to 428 mAh g^−1^, indicating the robust structure of NPC@MoS_2_/MXene. Figure 3f compares the capacity retention of NPC@MoS_2_, MoS_2_/MXene, and NPC@MoS_2_/MXene at different current densities, revealing the remarkable rate capability of NPC@MoS_2_/MXene. Moreover, as the current densities increased, the discharge/charge profiles of NPC@MoS_2_/MXene gradually exhibited a linear‐like sodiation/desodiation process, as shown in Figure 3g, indicating a pseudocapacitance‐controlled discharge/charge mechanism. Figure 3h provides a comparison of the rate performance among the prepared NPC@MoS_2_/MXene and other highly reported MoS_2_‐based electrodes for SIBs,^[^ ^23^ ^]^ demonstrating that NPC@MoS_2_/MXene exhibits excellent rate performance comparable to or even surpassing most reported electrodes. A more detailed comparison of SIBs performance of NPC@MoS_2_/MXene and other related electrodes is illustrated in Table S2 (Supporting Information), further highlighting the remarkable Na^+^ storage ability of the NPC@MoS_2_/MXene. Improved rate capability in electrodes is generally associated with enhanced charge transfer efficiency. Therefore, the charge transfer resistance of the electrodes was evaluated using Nyquist plots in Figure 3i. The NPC@MoS_2_/MXene exhibited the lowest charge transfer resistance (215 Ω, according to the fitting results) which is much lower than that of NPC@MoS_2_ and MoS_2_/MXene (453 and 306 Ω), indicating a synergistic effect among NPC, MXene, and MoS_2_, which facilitated transport kinetics and charge transfer efficiency.

**Figure 4:**
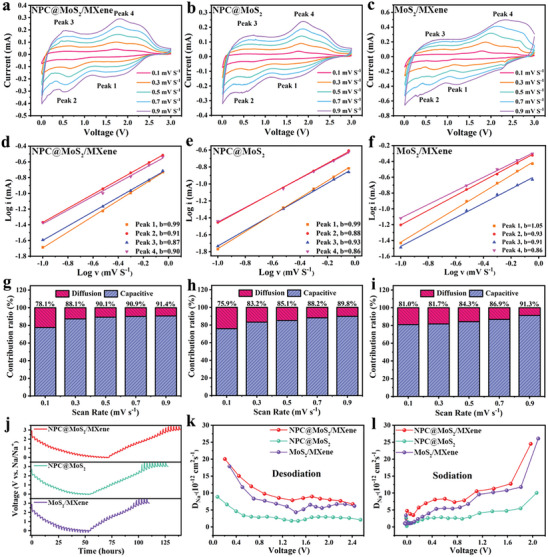
Sodium‐ion storage kinetics analysis of the NPC@MoS_2_/MXene, NPC@MoS_2_, and MoS_2_/MXene and, a–c) CV curves at different scan rates, d–f) *b*‐values based on lg*i* versus lg*v* plots, g–i) capacitive contribution at various scan rates, j) GITT curves and the corresponding calculated Na^+^ diffusion coefficient for k) desodiation and l) sodiation process.

Further investigation of the electrochemical kinetics of the electrodes was carried out using CV curves at different scan rates ranging from 0.1 to 0.9 mV s^−1^ (**Figure** **4**a–c). The rectangular CV shapes of NPC@MoS_2_, MoS_2_/MXene, and NPC@MoS_2_/MXene suggested a high proportion of capacitive behavior during their sodiation/desodiation process. Four peaks were selected to monitor the discharge/charge process. The coexistence of capacitive and diffusion behaviors was quantitatively analyzed using the equation *i*  =  *av^b^*, where *i* and *v* represent the current density and scan rate, respectively.^[^ ^24^ ^]^ The *b* value, calculated from the slope of log(*i*) versus log(*v*), reflects the ion storage behavior during the electrochemical process. A *b* value approaching 0.5 indicates diffusion‐controlled charge transfer, while a *b* value approaching 1.0 indicates pseudocapacitive behavior. Clearly, all three electrodes exhibited *b* values close to 1.0, indicating a capacitive‐dominated process during cycling (Figure 4d–f). Notably, the capacitive behavior in MoS_2_/MXene primarily originated from the high ionic conductivity and rich hydroxide groups in MXene, which serve as redox sites for charge transfer. Comparatively, although the surface area of NPC@MoS_2_/MXene was lower than that of NPC@MoS_2_, the introduction of MXene layers on the outer surface facilitated redox reactions, resulting in a more pronounced capacitive‐dominated process. Therefore, it is not surprising that the NPC@MoS_2_/MXene sample exhibited higher *b* values than NPC@MoS_2_, particularly at high current densities. The capacitance contribution of each electrode was further calculated using the equation *i*  = *k*_1_ *v* + *k*_2_ *v* ^1/2^, where *k_1_v* represents the surface capacitance effect and *k_2_v^1/2^* represents the diffusion‐controlled process.^[^ ^24^ ^]^ As shown in Figure 4g–i, as the scan rate increased, the capacitance contribution gradually increased as well. The highest pseudocapacitive ratio of 91.4% was achieved for the NPC@MoS_2_/MXene sample at a scan rate of 0.9 mV s^−1^. Therefore, at high current densities, the sodiation/desodiation process of NPC@MoS_2_/MXene was predominantly controlled by pseudocapacitive contributions, leading to excellent reversibility and rate capability.

Galvanostatic intermittent titration technique (GITT) was employed to investigate the electrochemical kinetics of the tested samples. GITT tests were conducted at a current density of 100 mA g^−1^ for 30 min, with a rest time of 120 min during the discharge/charge process. The chemical diffusion coefficient of sodium ions ($D_{Na^{+}}$) was determined using the following equation: ^[^ ^10b^ ^]^

(1) $$D_{Na^{+}}=\frac{4}{\pi \tau }\;{\left(\frac{m_{B}V_{m}}{M_{B}S}\right)}^{2}{\left(\frac{\Delta E_{s}}{\Delta E_{\tau }}\right)}^{2}$$

Here, τ represents the duration time of the current, *m_B_, V_m_* and *M_B_* are the loading mass, molar volume, and molecular weight of the active material, respectively. *S* denotes the contact area of the electrolyte/electrode, while Δ*Es* and Δ*E_t_* represent the changes in steady‐state voltage after subtracting the IR drop and the total transient change in voltage during a single titration. Figure 4j shows that all three electrodes exhibited a similar trend. Compared to the NPC@MoS_2_ sample, the MoS_2_/MXene demonstrated a lower voltage fluctuation during both the sodiation and desodiation processes, indicating a reduced kinetic barrier. This observation confirms the positive effect of MXene outer layers in improving the diffusion kinetics of the electrode. The NPC@MoS_2_/MXene sample exhibited the fastest Na^+^ diffusion kinetics, attributed to the synergistic effect of MoS_2_, NPC, and MXene. As depicted in Figure 4k,l, the NPC@MoS_2_/MXene consistently exhibited the highest Na^+^ diffusion coefficients compared to MoS_2_/MXene and NPC@MoS_2_ throughout the entire sodiation/desodiation process.

**Figure 5:**
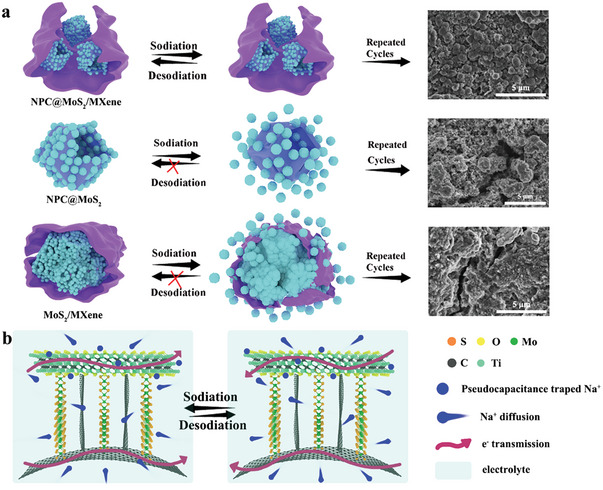
Illustration for a) Na^+^ storage process of the NPC@MoS_2_/MXene, NPC@MoS_2_, and MoS_2_/MXene and b) the structure superiority of the NPC@MoS_2_/MXene.

Based on the findings obtained from the electrochemical performance investigations, we have depicted a possible sodium storage process for the NPC@MoS_2_, MoS_2_/MXene, and NPC@MoS_2_/MXene electrodes in **Figure** **5**. In the case of the MoS_2_/MXene electrode, the high conductivity of MXene facilitates efficient utilization of MoS_2_. However, after repeated cycles, severe structural collapse and pulverization occur (as confirmed by the SEM image in the bottom row), resulting in unsatisfactory cycling stability. On the contrary, the presence of hollow NPC in the NPC@MoS_2_ sample acts as an inner support, anchoring the onion‐like MoS_2_ spheres and thereby improving cycling stability. The SEM image of the electrode after cycling shows enhanced structural integrity in the NPC@MoS_2_ compared to the MoS_2_/MXene sample. The NPC@MoS_2_/MXene sample exhibits the best electrochemical performance for several reasons. First, the MXene layer encapsulates the NPC@MoS_2_ units inside, creating high‐speed channels for rapid charge transfer and ion diffusion. Second, the dual‐reinforced structure, both internally with the NPC and externally with the MXene, provides the NPC@MoS_2_/MXene electrode with a robust framework capable of accommodating the repeated sodiation/desodiation process. Additionally, the exposed MXene surface in the NPC@MoS_2_/MXene, with its abundant surface functional groups, exhibits a high active surface‐redox pseudocapacitance behavior, particularly at high current densities.

**Figure 6:**
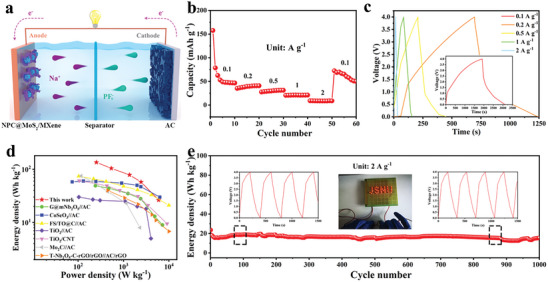
Electrochemical performance analysis of the assembled NPC@MoS_2_/MXene//AC SICs: a) schematic illustration, b) the rate performance and c) chronopotential potential curves of the SICs. d) Ragone plots of this SICs compared with other highly studied SICs. e) Cycling performance of this SICs at a current density of 2000 mA g^−1^. Inset (e) are the potential application and chronopotential potential curves at the different state, respectively.

The fast ion diffusion kinetics and long cycling stability of the NPC@MoS_2_/MXene make it a promising anode material for SICs. After presodiation at 100 mA g^−1^ for five cycles, the NPC@MoS_2_/MXene electrode was used as the anode for SICs assembling. A commercially purchased active carbon (AC) was used to fabricate the cathode. To assess the electrochemical performance of the commercial Activated Carbon (AC), half cells were assembled and tested within a voltage window ranging from 1 to 4 V, as shown in Figure S19 (Supporting Information). The observed rectangle‐like cyclic voltammetry (CV) shape of the commercial AC indicates a sodium storage performance dominated by pseudocapacitance, which is consistent with the discharge/charge plots. he discharge and charge capacities of the AC were calculated to be 166 and 149 mAh g^−1^, respectively. Consequently, the mass ratio between NPC@MoS_2_/MXene and AC was set at 1 to 3, and the voltage range for the Sodium‐Ion Capacitor (SIC) was established as 0.01 to 4.0 V. Specific capacity, power density, and energy density for this SIC were computed based on the combined weight of the cathode and anode. As illustrated in **Figure** **6**a, during the charging process, Na^+^ ions inserted into the NPC@MoS_2_/MXene anode, while FP_6_^−^ ions absorbed into the AC cathode. The discharge process is a reverse reaction to the charge process. Figure 6b showed that the as‐assembled SICs delivered high capacities of 49 mAh g^−1^ at a current density of 100 mA g^−1^. Even increased the current density to 1000 mA g^−1^, a high capacity of 21 mAh g^−1^ was still observed. Figure 6c showed that such remarkable capacity can be delivered within 160 s. The near‐linear curves in Figure 6c implied rapid ion storage kinetics of the SICs. The Ragone plot in Figure 6d shows that an ultrahigh energy density of 136 Wh kg^−1^ was achieved at a power density of 254 W kg^−1^. When the power density increased to 5940 W kg^−1^, the as‐assembled SICs could still deliver a high energy density of 27 Wh kg^−1^, which outperformed most of the reported SICs.^[^ ^25^ ^]^ Notably, the prepared SICs also showed a good cycling performance at a high current density of 2000 mA g^−1^ with a high energy density of 23 Wh kg^−1^ after 1000 cycles. An LED array can be easily lighted up, demonstrating the viability application of this SIC. The voltage versus time plots (inset Figure 6e) further confirmed the possible application of such SICs for other high power density devices. It should be noted that by replacing this commercial AC cathode with other more active materials the electrochemical performances of this NPC@MoS_2_/MXene‐based SICs may be further improved.

## 3 Conclusion

In summary, the prepared NPC@MoS_2_/MXene composite offers several key advantages: 1) Expanded interlayer distance of the MoS_2_ sheets, during the sulfidation process amorphous carbon layers can in situ intercalated into the MoS_2_ gallery. This intercalation provided sufficient channels for fast Na^+^ diffusion. 2) To protect the MoS_2_ layers from degradation during cycling, a hollow NPC polyhedron was used as an internal matrix to anchor the MoS_2_sheets, resulting in the formation of the NPC@MoS_2_ unit. 3) The NPC@MoS_2_ units were further encapsulated by a few‐layers of Ti_3_C_2_T_x_ MXene which introduced cross‐linked highly conductive pathways. Detailed characterizations demonstrated a synergistic coupling effect between NPC@MoS_2_ and MXene, leading to a robust electronic interaction that ensured the integrity of the target electrode. Moreover, the NPC@MoS_2_/MXene composite exhibited favorable capacitive behavior in redox reactions and excellent electrochemical reversibility due to its well‐designed structure. As a result, the prepared NPC@MoS_2_/MXene composite demonstrated superior electrochemical performances for both SIBs and SICs.

## 4 Experimental Section

### Synthesis of NPC@MoS_2_ and MoS_2_

60 mg of the fabricated NPC powders (synthesis process is given in the “Supporting information”) were dispersed into 20 mL ethanol, and sonicated for 10 min. To this solution, 0.3 g of polyvinylpyrrolidone (PVP) was added and further sonicated for 5 min, which was followed by another 30 min stirring. The PVP‐modified NPC was collected after centrifugation, wash, and dry. The resulting PVP‐modified NPC was added to a pyrrole solution (210 µL of pyrrole monomer dissolved in 12.5 mL ethanol) and stirred for 3 h (marked as solution A). 1.095 g phosphomolybdic acid (PMo_12_) and 40 mL ethanol solution was slowly added to solution A, The above mixture was rapidly stirred for 12 h and aged at room temperature for another 4 h. After centrifugation, washed and dry, the NPC@PPy‐PMo_12_ was obtained. Finally, a mixture of NPC@PPy‐PMo_12_ and S (mass ratio = 1:5) was transfer into a tube furnace and annealed with a ramp of 5 °C min^−1^ to 800°C for 2 h, under the protection of Ar atmosphere. After cooling to room temperature, the hollow NPC@MoS_2_ sample was obtained. The MoS_2_ sample was synthesized by directly adding phosphomolybdic acid solution to pyrrole solution without introducing PVP‐modified NPC, other conditions were similar to that of hollow NPC@MoS_2_.

### Synthesis of NPC@MoS_2_/MXene and MoS_2_/MXene

Monolayer MXene was prepared according to previous literature, and used directly.^[^ ^26^ ^]^ Synthesize processes of the NPC@MoS_2_/MXene were as following. First, 30 mg NPC@MoS_2_ was added into a Cetyl Trimethyl Ammonium Bromide solution (CTAB, 0.01 g CTAB and 20 mL ethanol), stirred for 6 h, and centrifugation to collect the CTAB modified NPC@MoS_2_. Second, the modified NPC@MoS_2_ was added to 30 mL monolayer MXene solution (1 mg mL^−1^) and stirred for 12 h. Finally, after centrifugation and vacuum drying at 60 °C for 12 h, the final NPC@MoS_2_/MXene sample was obtained. The MoS_2_/MXene was prepared by replacing NPC@MoS_2_ with MoS_2,_ other conditions were similar to that of hollow NPC@MoS_2_/MXene.

### Structural Characterization

Structural information of the prepared‐samples were carried out by X−ray diffraction (XRD, Bruker/D8 Advanced, Cu Kα radiation), transmission electron microscopy (TEM, JEOL JEM−2100F), field−emission scanning electron microscopy (FESEM, JEOL, SU8010), X−ray photoelectron spectroscopy (XPS, ThermoFisher, K−Alpha), Fourier transform infrared spectroscopy (FTIR, Nicolet iS10), Raman Spectrometer (Horiba scientific−LabRAM HR evolution with a 532 nm excitation light source), and nitrogen isotherms (Quantachrome, Autosorb−IQ2−VP).

### Fabrication of SIBs and SICs

The electrochemical performances of samples were tested by assembling CR2032 coin cells. Typically, the working electrodes were fabricated with 70 wt.% active materials, 20 wt.% carbon black, and 10 wt.% poly(vinyl difluoride) dissolved in N‐methyl‐2‐pyrrolidinone to form a slurry. The slurry was mix with a mortar‐pestle and then coated onto a Cu foil with 100 mm thickness. After vacuum drying at 80 °C for 12 h, the electrodes was cut into 8 mm disks with active material loading mass of ≈1.5 mg cm−2. Fresh sodium disks were used as both counter and reference electrodes. Whatman glass fiber was used as separator. 1 m NaFP6 in ethylene carbonate/dimethyl carbonate/ethyl‐methyl carbonate (1:1:1 by volume) with 5 wt.% fluoroethylene carbonate as the electrolyte. The specific capacity of electrodes was calculated based on the whole mass of active materials. As for the SICs, the Hollow NPC@MoS2/MXene anodes were presodiated at a current density of 100 mA g−1 for five cycles, a commercial purchased active carbon (AC, PEC‐04) was used as cathode, the separator and electrolyte were the same as those for the SIBs half‐cells.

### Electrochemical Characterization

Galvanostatic charge–discharge tests were carried out at room temperature on a battery testing system (LAND Wuhan, China). Cyclic voltammetry (CV) tests and electrochemical impedance spectroscopy (EIS) were carried out on a CHI660E electrochemical workstation. Cyclic voltammetry curves were tested from 0.01 to 3.0 V with a scan rate of 0.1–0.9 mV s^−1^. The EIS profiles were measured within a frequency ranging from 100 kHz to 10 mHz.

## Conflict of Interest

The authors declare no conflict of interest.

## Supporting information

Supporting Information

## Data Availability

Research data are not shared.
