# Supplementary material for: Hollow Carbon and MXene Dual‐Reinforced MoS2 with Enlarged Interlayers for High‐Rate and High‐Capacity Sodium Storage Systems
Source: Adv Sci (Weinh). 2024 Jan 22;11(37):2400364. doi: 10.1002/advs.202400364 (PMC11462304; doi:10.1002/advs.202400364)
Supplement: Supplementary file 1 — Supporting Information [file ADVS-11-2400364-s001.pdf]

ADVANCED SCIENCE
Open Access

Supporting Information

for *Adv. Sci.*, DOI 10.1002/advs.202400364

Hollow Carbon and MXene Dual-Reinforced MoS_2 with Enlarged Interlayers for High-Rate and High-Capacity Sodium Storage Systems

Hanqing Pan, Yan Huang, Xinnuo Cen, Ming Zhang, Jianhua Hou, Chao Wu, Yuhai Dou, Bing Sun, Ying Wang, Binwei Zhang* and Lei Zhang**

Supporting information

Hollow Carbon and MXene Dual-Reinforced MoS_2 with Enlarged Interlayers for High-Rate and High Capacity Sodium Storage Systems

Hanqing Pan, Yan Huang, Xinnuo Cen, Ming Zhang, Jianhua Hou, Chao Wu, Yuhai Dou, Bing Sun, Ying Wang, Binwei Zhang,* Lei Zhang,**

H. Q. Pan, Y. Huang, X. N. Cen, M. Zhang, Prof. Y. Wang

Jiangsu Key Laboratory of Green Synthetic Chemistry for Functional Materials,

School of Chemistry & Materials Science, Jiangsu Normal University, Xuzhou, Jiangsu 221116, PR China.

E-mail: yingwang@jsnu.edu.cn

Prof. B. Zhang

School of Chemistry and Chemical Engineering, Chongqing University, Chongqing 401331, PR China.

Center of Advanced Electrochemical Energy, Institute of Advanced Interdisciplinary Studies, Chongqing University, Chongqing 401331, PR China.

E-mail: binwei@cqu.edu.cn

Dr. L. Zhang

Centre for Catalysis and Clean Energy, Gold, Coast Campus, Griffith University, Gold Coast, QLD 4222, Australia.

E-mail: lei.zhang@griffith.edu.au

Prof. J. Hou

College of Environmental Science and Engineering, Yangzhou University, Yangzhou, Jiangsu 225009, China

Prof. C. Wu, Prof. Y. H. Dou

Institute of Energy Materials Science, University of Shanghai for Science and Technology, Shanghai 200093, China

Dr. B. Sun

Centre for Clean Energy Technology, School of Mathematical and Physical Sciences, Faculty of Science, University of Technology Sydney, Ultimo NSW 2007, Australia

Experimental Section:

Materials Fabrications

Synthesis of hollow nitrogen-doped carbon (HNC): 0.525 g anhydrous zinc acetate and 0.789 g 2-methylimidazole were added to 60 mL methanol, respectively. The above solution was mixed together, stirred for 5 min, and then aged at room temperature for 24 hours. After centrifugation, wash, and drying, ZIF-8 powders were collected. 0.6 g tannic acid was dissolved in 40 mL ethanol, and then the prepared ZIF-8 was added to the above solution, followed by ultrasonication for 5 minutes. After centrifugation, washed with ethanol, and dried at 60°C for 12 hours, hollow ZIF-8 was collected. The hollow ZIF-8 was further transferred into a tube furnace and annealed under Ar atmosphere at 800°C for 2 hours, with a heating rate of 5°C min^{-1}. After naturally cooling into room temperature the hollow N-doped carbon (HNC) powders were collected.

Calculations:

The specific capacitance (C, F g^{-1}), energy density (E, Wh kg^{-1}) and power density (P, W kg^{-1}) are calculated through the following equations ground on the GCD tests:

$$C = It / (\Delta V \times m) \quad (1)$$

$$E = C\,(V_{max}^2 - V_{min}^2) / (2 \times 3.6) \quad (2)$$

$$P = E \times 3600 / t \quad (3)$$

Where I is the discharge current (A), t is the discharge time (s), ΔV is the potential change, m is the total mass of active materials in the anode and cathode (g), and V_{max} and V_{min} are the maximum and minimum working voltage (V).

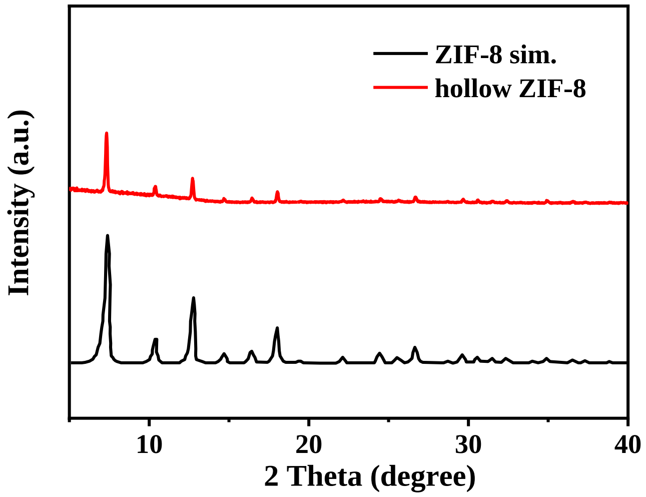

Figure S1. XRD patterns for the as-prepared ZIF-8 and hollow ZIF-8.

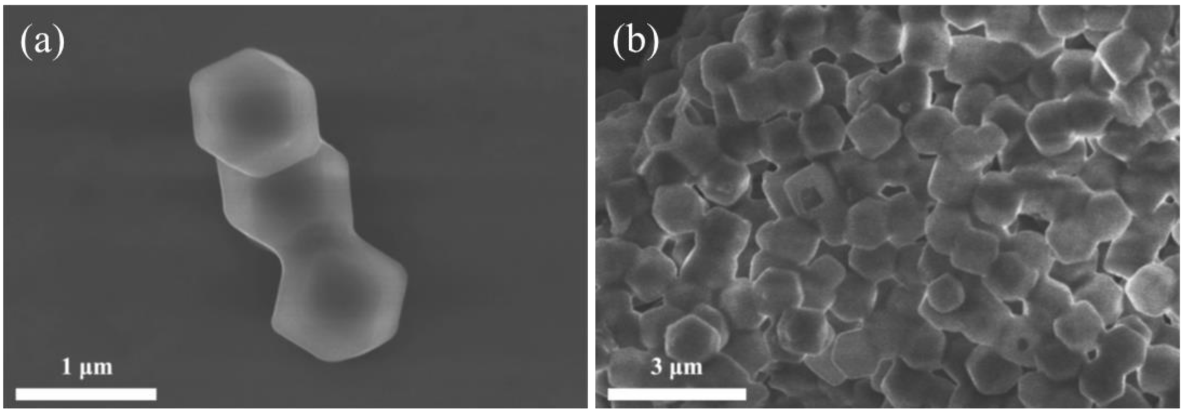

Figure S2. SEM images of (a) the ZIF-8, (b) the hollow ZIF-8.

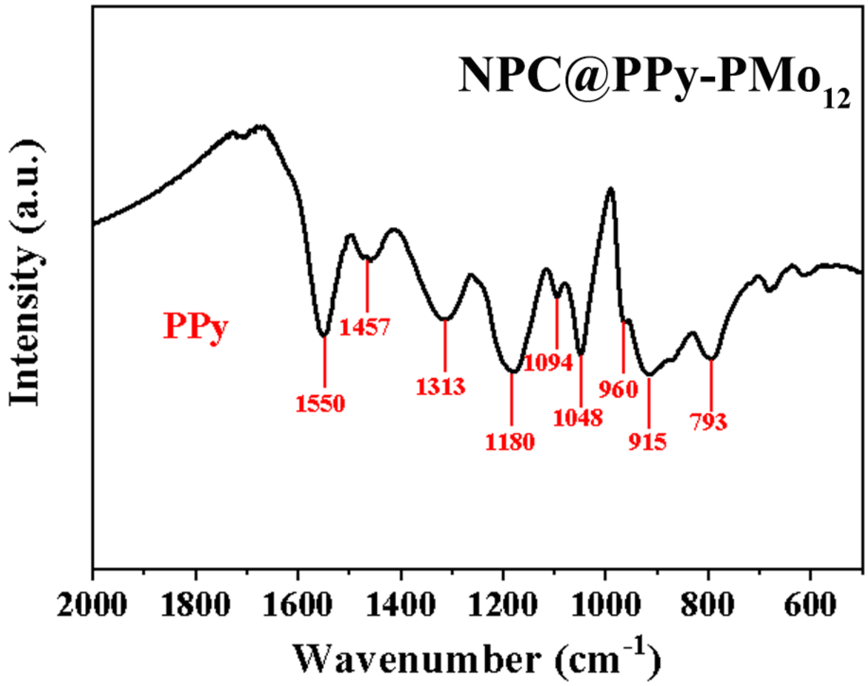

Figure S3. FTIR plots for the NPC@PPy-PMo_{12} sample.

The peak at 1550 cm^{-1} is caused by the C=C stretching vibration of pyrrole ring. The peak at 1457 cm^{-1} is attributed to the stretching vibration of C-N bond in the pyrrole ring. The peak at 1298 cm^{-1} is related to the in-plane vibration of C-H bond. The characteristic peak at 1180 cm^{-1} corresponds to the bending stretching mode of H in the N-pyrrole ring. The characteristic peak at 1048 cm^{-1} is related to the in-plane stretching vibration of the C-H and N-H bonds, and the characteristic peak at 918 cm^{-1} corresponds to the stretching vibration of the C-C bond. Existence of above bonds confirming the successful coprecipitation of PPy@PMo_{12}.

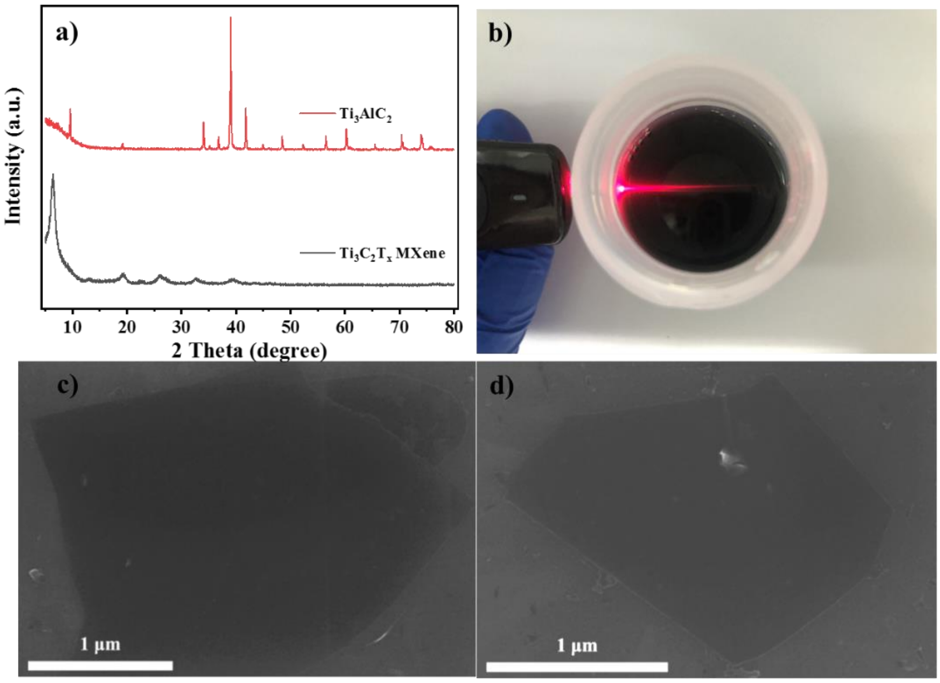

Figure S4. (a) XRD patterns of the Ti_3AlC_2 MAX phase and the Ti_3C_2Tx MXene phase, (b) optical image of the MXene solution, (c, d) SEM images of a monolayer MXene in different magnifications.

After HF etching, the intense peaks for Ti_3AlC_2 disappears. The (002) peak of the prepared MXene shifts to a lower angle, indicating complete transformation of Ti_3AlC_2 to $Ti_3C_2T_x$. Furthermore, the number and intensity of the $Ti_3C_2T_x$ MXene peak was reduced, indicating a thinner layered structure of the prepared Ti_3C_2Tx MXene. An obvious Tyndall effect was observed in the black MXene solution further confirms the successful synthesis of thinner layered MXene. SEM image of MXene showed a smooth surface and an average length of 2-3 μm.

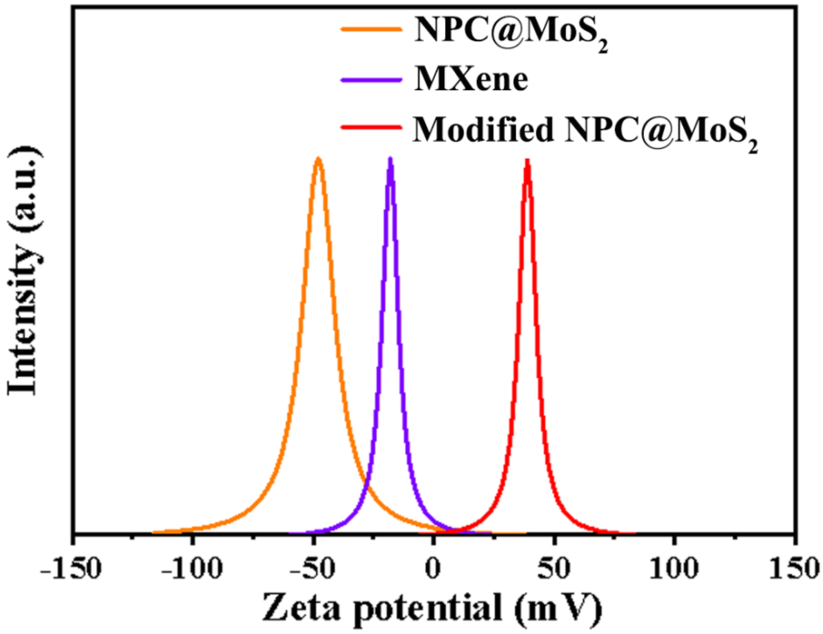

Figure S5. Zeta potential test of the pure MXene, NPC@MoS_2, and the CTAB modified NPC@MoS_2.

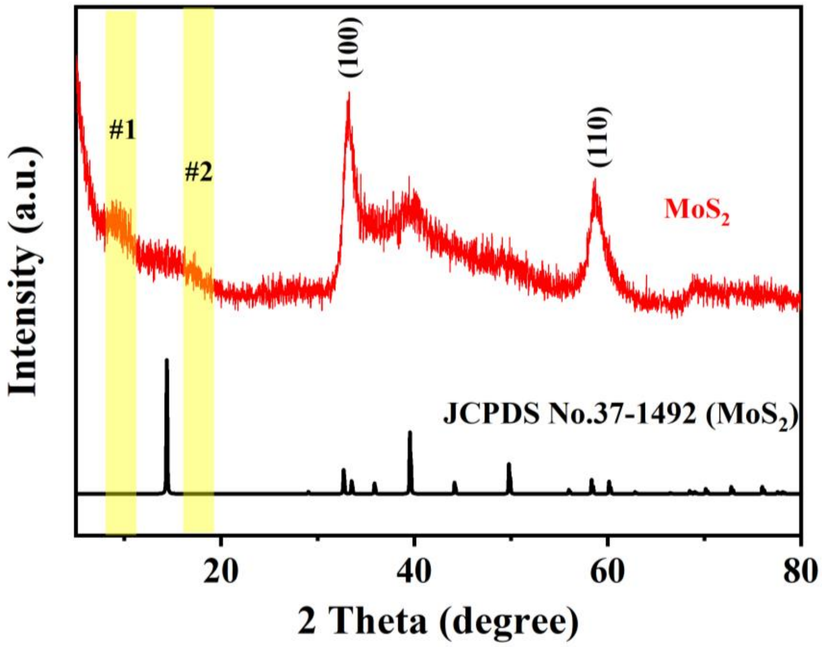

Figure S6. XRD pattern of pure MoS_2.

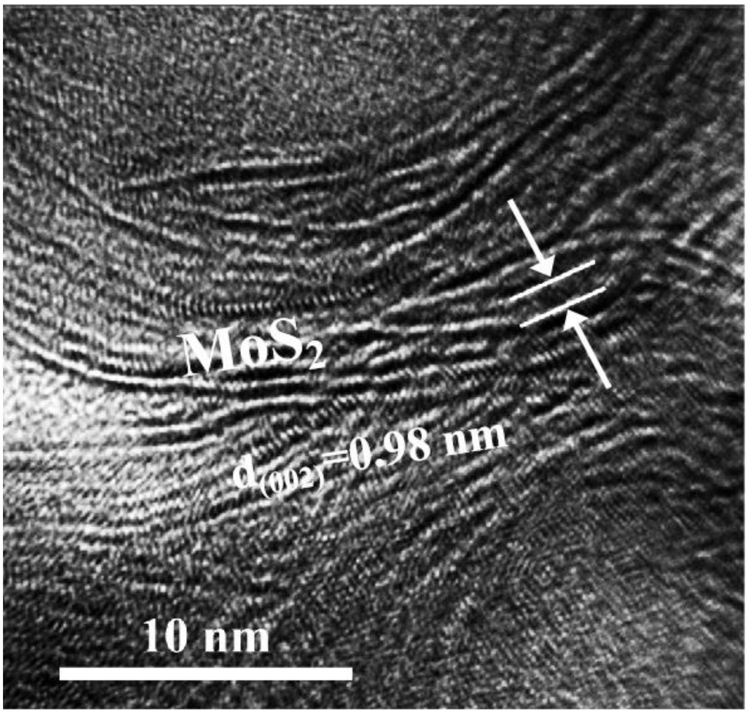

Figure S7. HR-TEM image of pure MoS_2

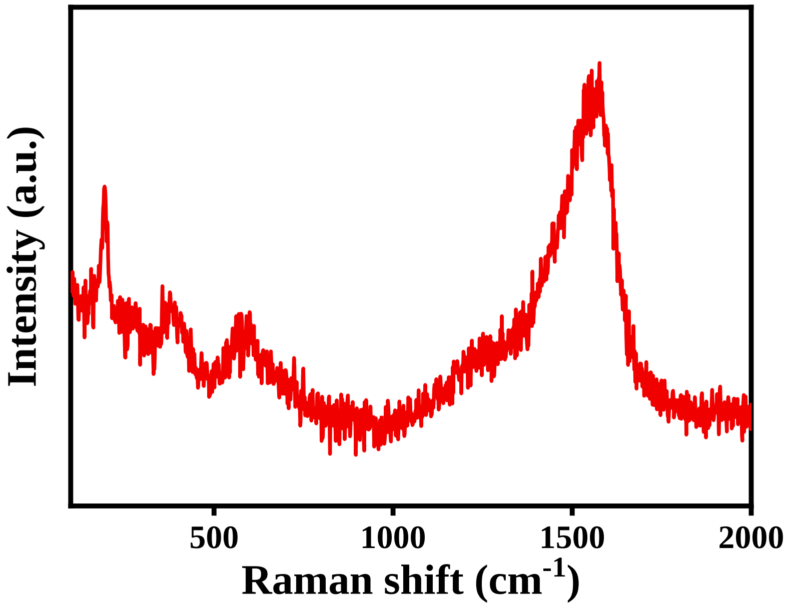

Figure S8. Raman plot of the pure MXene.

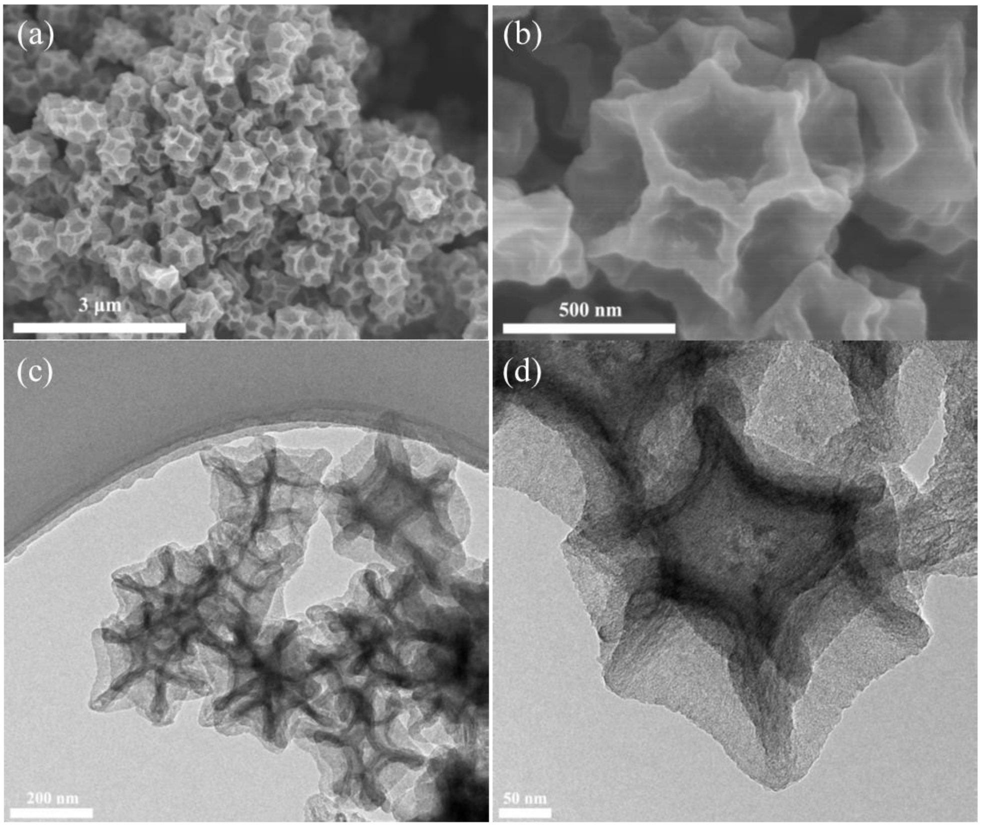

Figure S9. (a, b) SEM and (c, d) TEM images of the hollow NPC in different magnifications.

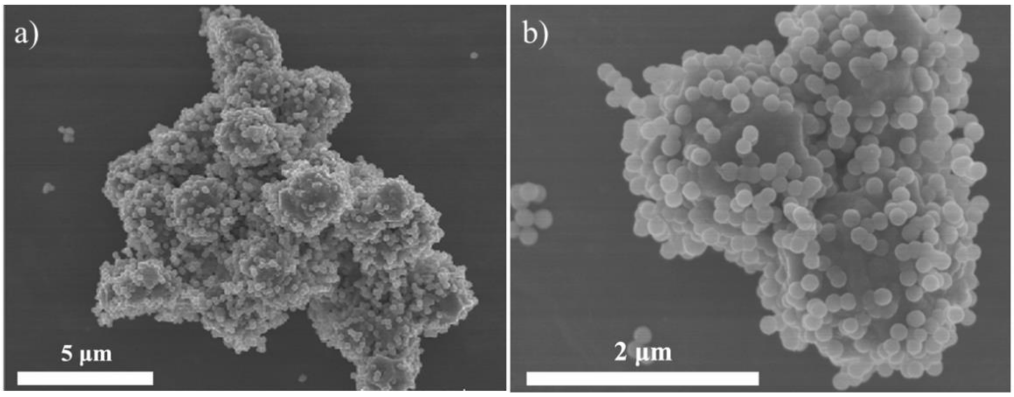

Figure S10. (a, b) SEM images of the NPC@PPy-PMo_{12} in different magnifications.

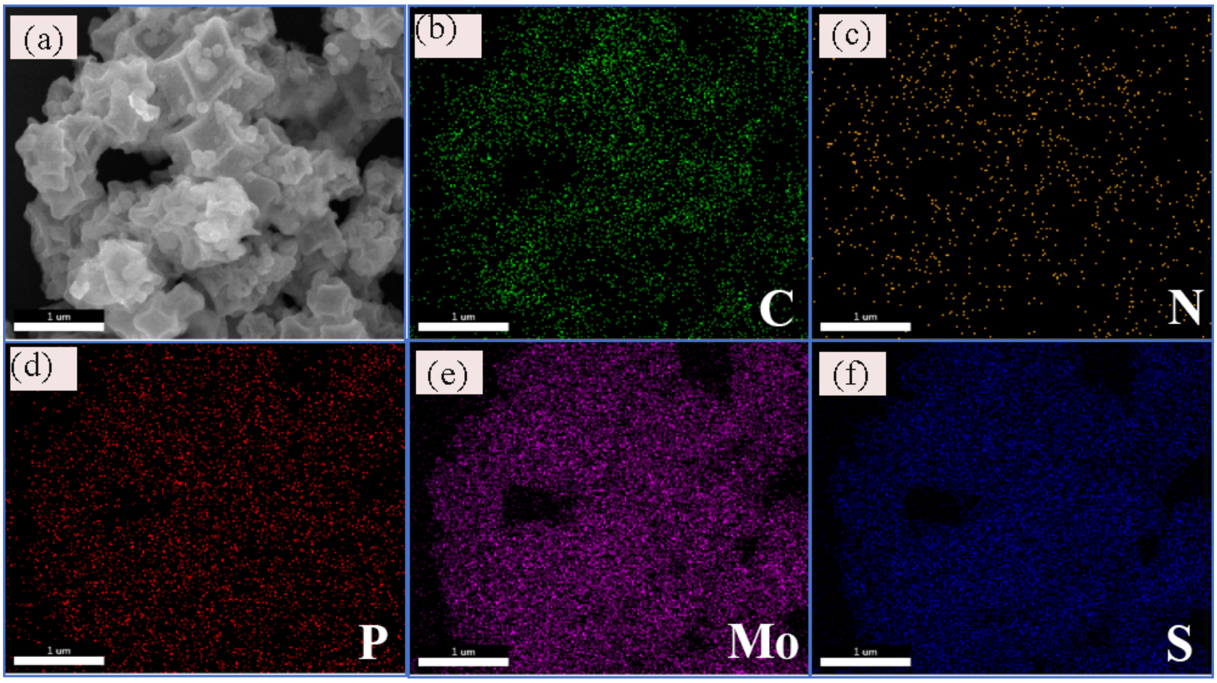

Figure S11. (a) SEM image, and EDS mapping of the (b) C, (c) N, (d) P, (e) Mo, and (f) S for the NPC@MoS_2.

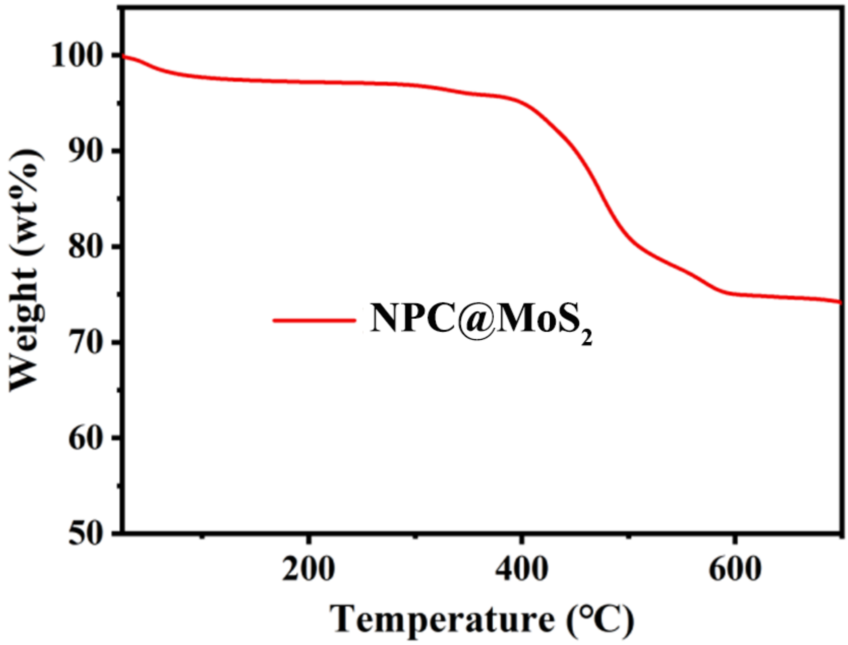

Figure S12. TGA curve of NPC@MoS_2 in air at a heating rate of 10 ˚C min^{-1}.

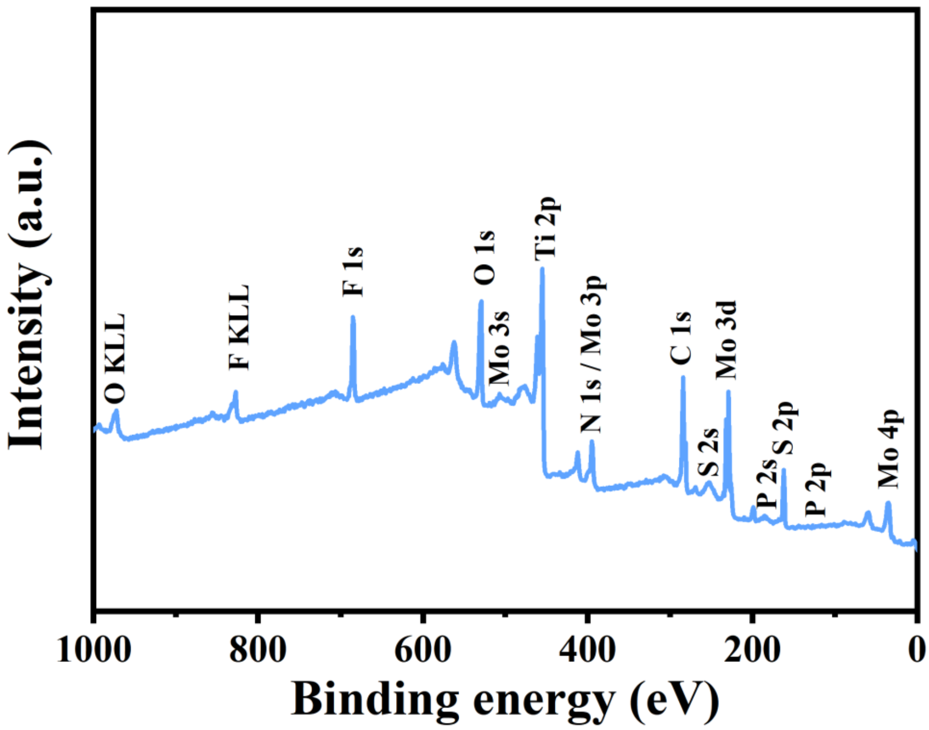

Figure S13. XPS survey of the NPC@MoS_2/MXene.

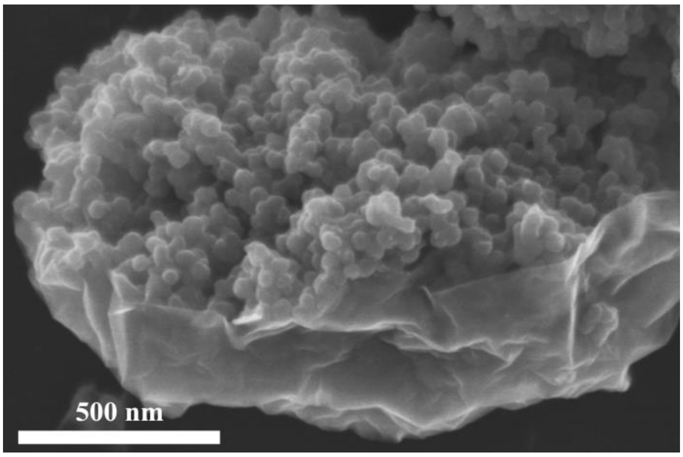

Figures S14. SEM image of the MoS_2/MXene.

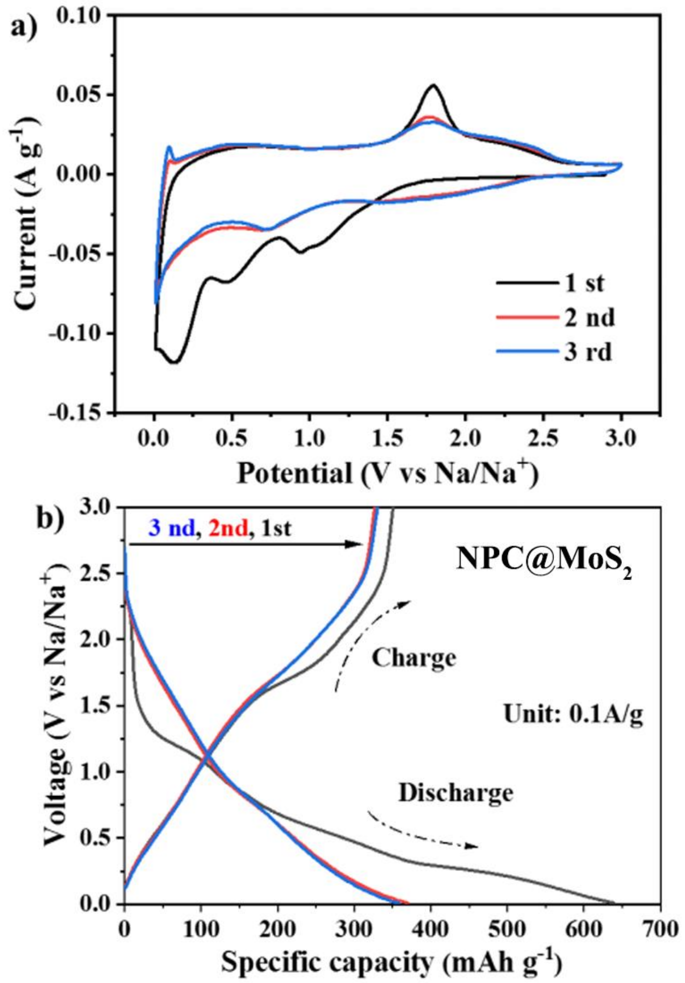

Figure S15. Electrochemical performances of the NPC@MoS_2 for SIBs. (a) CV curves at a scan rate of 0.1 mV s^{-1}, (b) discharge-charge plots at 100 mA g^{-1}.

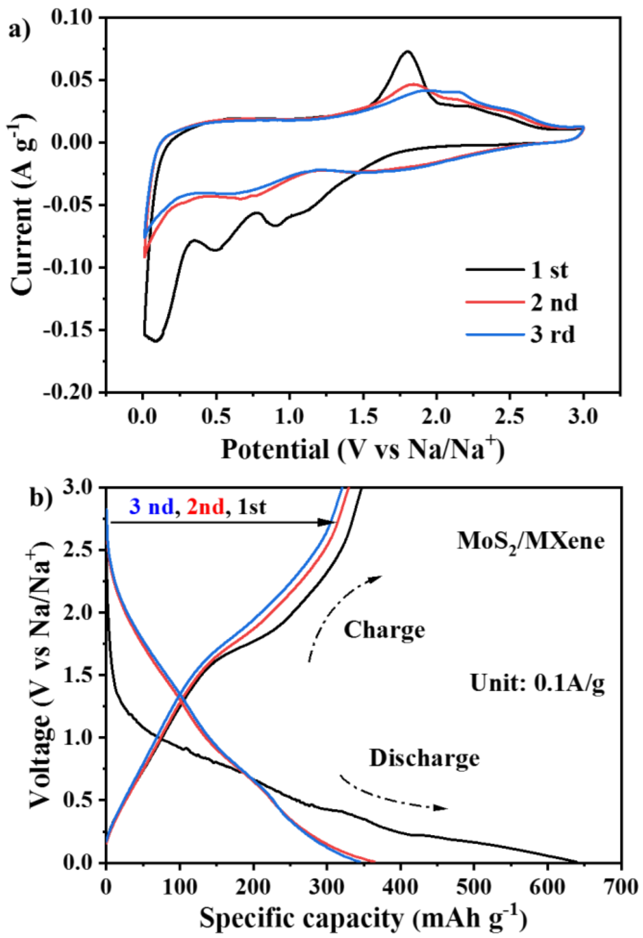

Figure S16. Electrochemical performances of the MoS_2/MXene for SIBs. (a) CV curves at a scan rate of 0.1 mV s^{-1}, (b) discharge-charge plots at 100 mA g^{-1}.

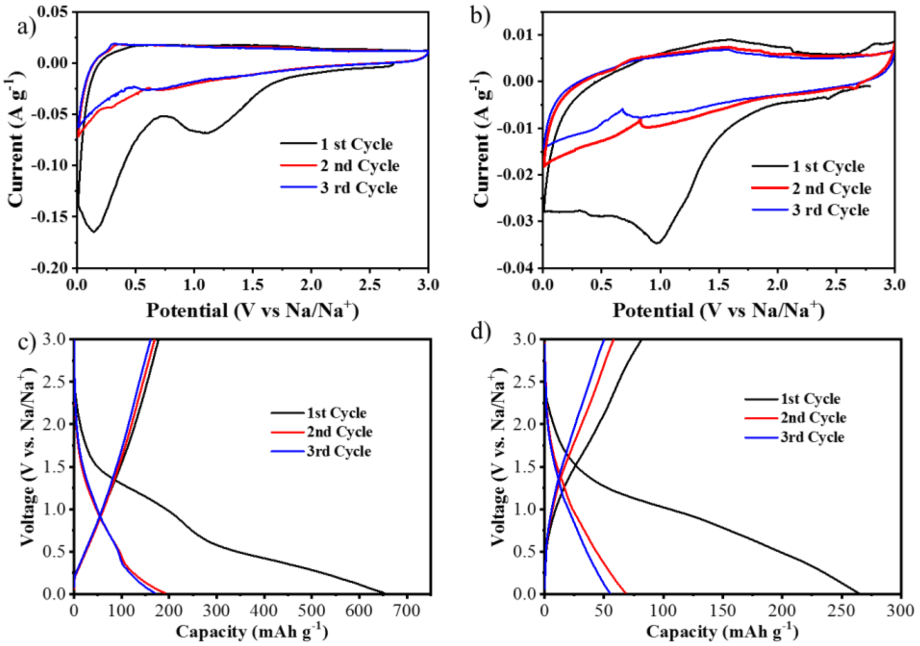

Figure S17. CV curves of (a) the NPC and (b) MXene at a scan rate of 0.1 mV s^{-1}. Discharge/charge plots of the (c) NPC and (d) MXene at a current density of 100 mA g^{-1}.

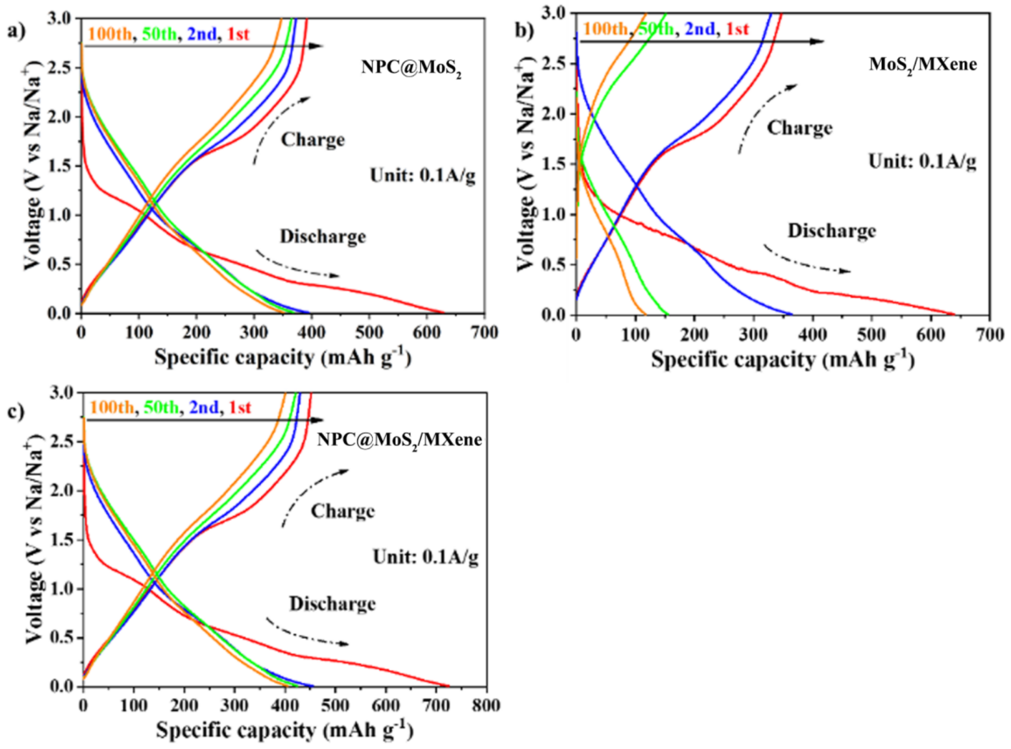

Figure S18. Discharge/charge plots of the (a) NPC@MoS_2, (b) MoS_2/MXene, (c) NPC@MoS_2/MXene at a current density of 100 mA g^{-1}.

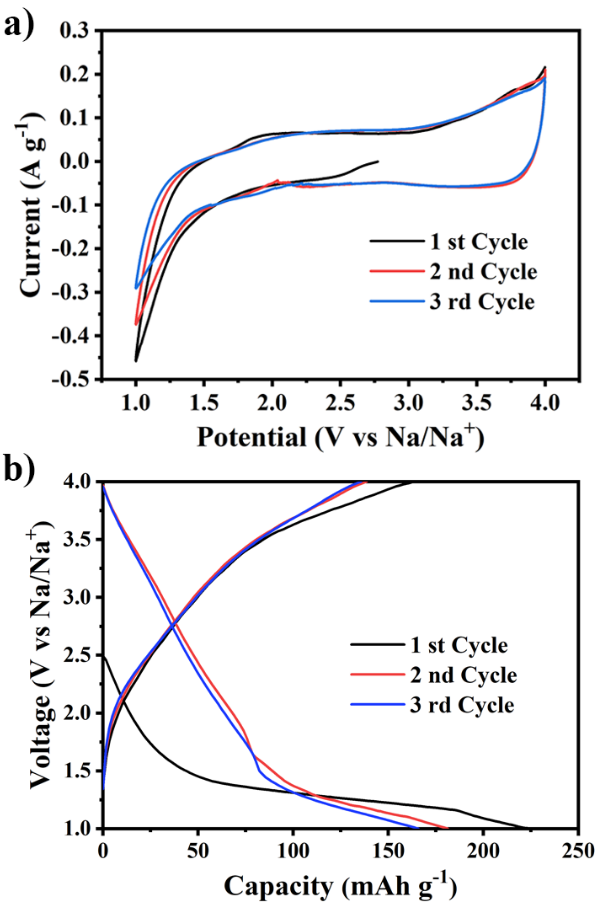

Figure S19. (a) CV curve of the Commercial AC at a scan rate of 0.5 mV s^{-1}. (b) Discharge/charge plots of the Commercial AC at a current density of 100 mA g^{-1}.

Table S1. Chemical composition in NPC@MoS_2, MoS_2/MXene and NPC@MoS_2/MXene*.

**Name**	**NPC (wt.%)**	**MoS_2 (wt.%)**	**MXene (wt.%)**
NPC@MoS_2	13.5	86.5	---
MoS_2/MXene	---	50	50
NPC@MoS_2/MXene	6.8	43.2	50

*The chemical composition in each sample was calculated based on the TGA curves in Figure S12, and the added MXene during the synthesis process.

Table S2. Electrochemical comparison of the MoS_2-based electrodes for SIBs

**Sample name**	**Current (Ah/g)**	**Cycle number**	**Capacity (mAh g^{-1})**	**Ref.**
**NPC@MoS_2/MXene**	**1.0**	**1000**	**279**	**This work**
MoS_2/NGA1	0.1	80	673	1
1T−MoS_2@rGO	1.0	100	280	2
US−MoS_2@NG	1.0	1000	198	3
MoS_2/graphene	0.3	250	421	4
MoS_2/C@RGO	1.0	300	257	5
MoS_2@LEGr	0.3	100	377	6
v−MoS_2/rGO	2.0	1000	251	7
GR@MoS_2@PG	1.0	1000	200	8
1T MoS_2	0.5	200	313	9
MoS_2/graphene	0.5	200	317	10
MoS_2/$Ti_3C_2T_x$@NC	0.5	500	207	11
F−MoS_2@NCN−0.8	1.0	1000	256	12
P−MoS_2@C/CNTP	1.0	1200	249	13
MoS_2/TSFC-2.5-180-12	0.1	500	243	14
S-BC/E-MoS_2@N-C	0.2	200	371	15
MoS_2/MXene	1.0	900	326	16

Reference:

[1] J. Yuan, J. Zhu, R. H. Wang, Y. X. Deng, S. Zhang, C. Yao, Y. J. Li, X. L. Li, C.

H. Xu, *Chem. Eng. J.* **2020**, *398*, 125592.
[2] X. L. Yu, R. X. Li, X. Y. Hu, R. He, K. H. Xue, R. R. Sun, T. Yang, W. L. Wang, X. Fang, *J. Solid State Chem.* **2021**, *297*, 122027.
[3] X. Xu, R. S. Zhao, W. Ai, B. Chen, H. F. Du, L. S. Wu, H. Zhang, W. Huang, T. Yu, *Adv. Mater.* **2018**, *30*, 1800658.
[4] D. Sun, D. L. Ye, P. Liu, Y. G. Tang, J. Guo, L. Z. Wang, H. Y. Wang, *Adv. Energy Mater.* **2018**, *8*, 1702383.
[5] Y. F. Li, H. J. Mao, C. Zheng, J. J. Wang, Z. Z. Che, M. D. Wei, *J. Phys. Chem. Solids* **2020**, *136*, 109163.
[6] J. H. Li, H. K. Wang, W. Wei, L. J. Meng, *Nanotechnology* **2019**, *30*, 104003.
[7] H. Li, X. Z. Wen, F. Shao, S. W. Xu, C. Zhou, Y. F. Zhang, H. Wei, N. T. Hu, *J. Alloy. Compd.* **2021**, *877*, 160280.
[8] X. Q. Hao, Z. Q. Jiang, X. N. Shang, X. N. Tian, X. P. Chen, ; X. G. Hao, Z.-J. Jiang, *J. Alloy. Compd.* **2020**, *845*, 155336.
[9] X. M. Geng, Y. C. Jiao, Y. Han, A. Mukhopadhyay, L. Yang, H. L. Zhu, *Adv. Funct. Mater.* **2017**, *27*, 1702998.
[10] L. F. Fei, M. Xu, J. Jiang, S. M. Ng, L. L. Shu, L. Sun, K. Y. Xie, H. T. Huang, C. W. Leung, C. L. Mak, Y. Wang, *RSC Adv.* **2018**, *8*, 2477.
[11] J. B. Li, S. C. Tang, Z. Q. Li, C. Y. Wang, J. L. Li, X. D. Li, Z. B. Ding, L. K. Pan, *ChemSusChem* **2021**, *14*, 5293.
[12] J. F. Li, W. X. Gao, L. Y. Huang, Y. C. Jiang, X. T. Chang, S. B. Sun, L. K. Pan, *Appl. Surf. Sci.* **2022**, *571*, 151307.
[13] S. M. Sui, H. N. Xie, M. Liang, B. C. Chen, C. Y. Liu, E. Z. Liu, B. Chen, L. Y. Ma, J. W. Sha, N. Q. Zhao, *Adv. Funct. Mater.* **2022**, *32*, 2110853.
[14] Y. Luo, X. N. Li, X. Y. Hao, Y. Y. Xu, S. W. Tang, K. Zhang, A. M. Qin, *J. Energy Storage* **2023**, *67*, 107463.
[15] H. Huang, L. J. Zhao, Z. Q. Zheng, D. Xie, P. Liu,; Y. J. Mai, F. L. Cheng, *Electrochim. Acta* **2023**, *461*, 142626.
[16] J. C. Han,; W. B. Xu, Z. M. Liu, Z. Y. Gao, S. Tao, H. H. Min, H. Yang, J. Wang, *J. Alloy. Compd.* **2023**, *957*, 170282.
